# Intracellular Metabolites in Marine Microorganisms during an Experiment Evaluating Microbial Mortality

**DOI:** 10.3390/metabo10030105

**Published:** 2020-03-12

**Authors:** Krista Longnecker, Elizabeth B. Kujawinski

**Affiliations:** Woods Hole Oceanographic Institution, Department of Marine Chemistry and Geochemistry, Woods Hole, MA 02543, USA; ekujawinski@whoi.edu

**Keywords:** marine microorganisms, intracellular metabolites, dilution experiment, compatible solutes, Weighted Correlation Network Analysis

## Abstract

Metabolomics is a tool with immense potential for providing insight into the impact of biological processes on the environment. Here, we used metabolomics methods to characterize intracellular metabolites within marine microorganisms during a manipulation experiment that was designed to test the impact of two sources of microbial mortality, protozoan grazing and viral lysis. Intracellular metabolites were analyzed with targeted and untargeted mass spectrometry methods. The treatment with reduced viral mortality showed the largest changes in metabolite concentrations, although there were organic compounds that shifted when the impact of protozoan grazers was reduced. Intracellular concentrations of guanine, phenylalanine, glutamic acid, and ectoine presented significant responses to changes in the source of mortality. Unexpectedly, variability in metabolite concentrations were not accompanied by increases in microbial abundance which indicates that marine microorganisms altered their internal organic carbon stores without changes in biomass or microbial growth. We used Weighted Correlation Network Analysis (WGCNA) to identify correlations between the targeted and untargeted mass spectrometry data. This analysis revealed multiple unknown organic compounds were correlated with compatible solutes, also called osmolytes or chemical chaperones, which emphasizes the dominant role of compatible solutes in marine microorganisms.

## 1. Introduction

The actions of microbial-sized cells impact the composition of organic carbon found in marine ecosystems. In the oceans, small autotrophic organisms are generally a source of new organic compounds as they convert inorganic carbon dioxide into organic matter, and existing metabolomics research has provided insight into the molecular-level composition of the dissolved and particulate components of this organic matter e.g., [1,2]. At the same time, heterotrophic microorganisms use organic matter as energy and carbon sources, and this process consumes and alters organic matter e.g., [3,4]. These heterotrophic microorganisms are subsequently consumed by predators which transfers organic carbon to larger members of a marine food web. The vast size of the worlds’ oceans and the amount of carbon they hold makes these processes important components of the global carbon cycle.

Marine scientists regularly use manipulation experiments to quantify microbial processes within the marine carbon cycle and to characterize the organic carbon transferred within marine food webs. For example, manipulation experiments can be used to quantify rates for a specific biogeochemical process (e.g., carbon or nitrogen fixation) or to assess the diversity of microorganisms involved in a given biogeochemical process e.g., [5,6,7]. Metabolomics has the potential to inform manipulation experiments by providing the tools needed to characterize the organic compounds that are produced and consumed by marine microorganisms under different conditions. In this project, we take advantage of analytical developments that allow the use of mass spectrometry with marine samples [8]. We use these methods to assess the outcome of a manipulation experiment using seawater from the surface ocean. 

Mortality in marine microorganisms can be attributed to two processes: consumption by protozoan grazers or lysis of cells by viruses. One means to consider the impact of how a cell dies is to manipulate the microbial community in order to reduce the encounter rates between predators or viruses and their microbial prey. This experimental manipulation relies on the differences in size between microorganisms and their potential predators. Protozoan grazers are larger than most microorganisms, while viruses are smaller than microorganisms. Through careful selection of different filtration membranes, microorganisms in seawater can be separated from their predators. In this project we used seawater diluted with 0.2-µm filtered water to reduce levels of protozoan grazing and compared this to samples with reduced levels of viral lysis and protozoan grazing that were established by diluting seawater with 30-kDa filtered seawater. This method, broadly termed a dilution experiment, was originally proposed by Landry and Hassett [9] to examine protozoan grazing and was later modified to include viral lysis [10]. While these experiments are widely used, there are recognized biases regarding shifts in the diversity and activity of a microbial community during these experiments e.g., [11,12,13]. Furthermore, while we know a diverse set of organic compounds, or metabolites, exists within marine microbial cells [1,14], we have no information about the variability in these compounds during a dilution experiment. This information is critical to address how mortality may change the composition of organic carbon released by a microbial cell into the marine environment.

Here, we used targeted and untargeted metabolomics methods to assess short-term changes in intracellular organic compounds, or metabolites, in an incubation experiment established with seawater from the western Atlantic Ocean. Targeted metabolomics is quantification of known organic compounds while untargeted metabolomics is a more open-ended exploration of the organic compounds in a sample. Analytical and computational advances in metabolomics allow us to assess how organic compounds within microbial cells shift when the community is exposed to varying sources of mortality. We present our results in the context of marine science, while simultaneously considering the benefits and challenges facing marine metabolomics research.

## 2. Results

### 2.1. Environmental Data

**Figure 1:**
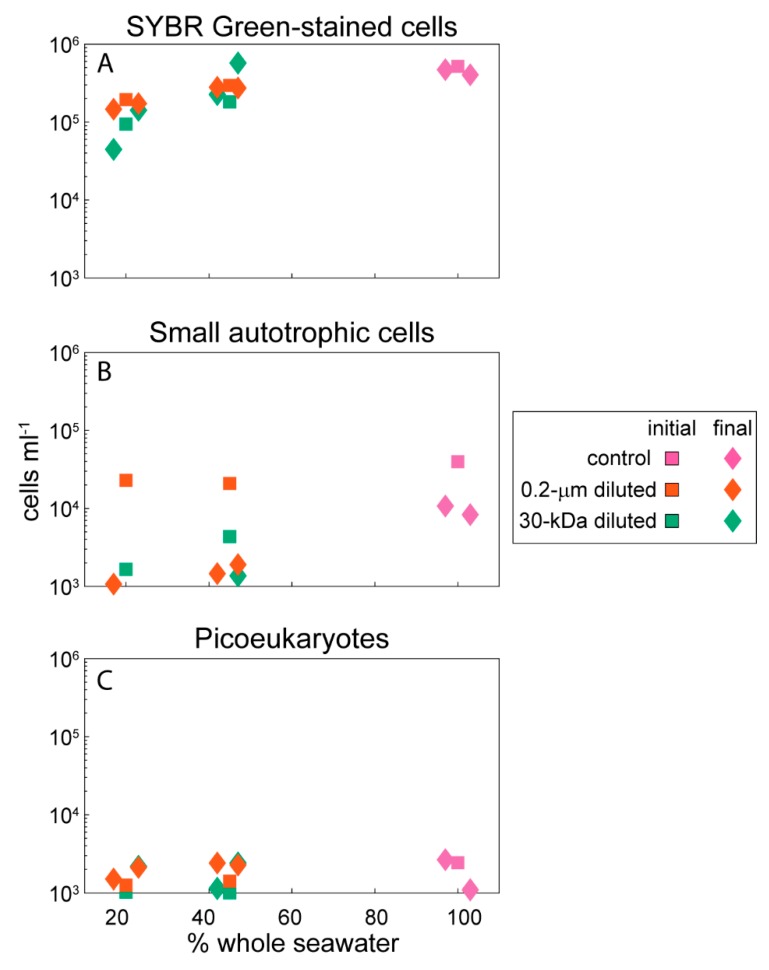
Abundance of microbial-sized cells at the initial and final points in the experiment. (**A**) SYBR Green-stained cells are heterotrophic cells counted with flow cytometry, (**B**) small autotrophic cells are the autofluorescing cells within the flow cytometry data, and (**C**) picoeukaryotes were counted with epifluorescence microscopy. The abundances have been jittered on the *x*-axis to improve clarity of the data presentation. The total abundance of microbial-sized cells was defined as the sum of all three datasets and was used to normalize the metabolite concentrations to cell-specific values for each organic compound. Data are plotted on a log scale to allow comparisons across the different groups.

The seawater used to establish the experiments was from the deep chlorophyll maximum located at 70 m below the surface. The water temperature was 27.6 °C, salinity was 36.3, and the total organic carbon (TOC) concentration of seawater sampled directly from the Niskin bottle was 73 µM. The total organic carbon concentrations within the incubation bottles ranged from 69–86 µM, with slight variability at the onset of the experiment and across the different experimental treatments (Table 1). Heterotrophic bacterial cells were the majority of the microbial-sized cells in this experiment (Figure 1A), with smaller contributions from small autotrophic cells that were primarily *Prochlorococcus* and *Synechococcus* (Figure 1B). The autotrophic cells counted by flow cytometry showed slight decreases in abundance over the 24-hour incubation, while the heterotrophic cells had a variable response in the 30-kDa filtered bottles with 45% whole seawater but otherwise showed no change in abundance. Picoeukaryote abundances based on epifluorescence microscopy showed increases in the 0.2-µm diluted treatments but a variable response across replicates from the controls and the 30-kDa diluted treatments (Figure 1C).

### 2.2. Targeted Metabolomics Data Results 

We only considered organic compounds in the targeted metabolomics method that were present above the detection limit in at least three samples. This reduced the set of quantifiable organic compounds from 70 to 24 compounds. Dividing the concentrations by the total abundance of microbial-sized cells captured by each filter allowed us to consider differences in cell-specific concentrations and removed the impact of different microbial abundances in each of the experimental manipulations (Supplemental Figure S1). There were no significant differences in the cell-specific concentrations of organic compounds between the bottles with 20% and 45% whole seawater; therefore, these two treatments are combined for all subsequent data analysis. In future experiments, we suggest triplicate bottles for each treatment in order to better utilize the statistical tools needed to assess differences across treatments. Four metabolites showed significant differences in the treatments (0.2-µm diluted, 30-kDa diluted) compared to the control at the final time point of the experiment (one-way ANOVA followed by Dunnett’s post hoc multiple comparisons test, Table 2). All four of these metabolites (guanine, glutamic acid, phenylalanine, and ectoine) showed significant differences between the control and the 30-kDa diluted treatments, but only ectoine and guanine were significantly different between the control and the 0.2-µm diluted treatments. The remaining 20 compounds did not show significant differences between the controls and the treatments with 0.2-µm diluted or 30-kDa diluted seawater.

**Figure 2:**
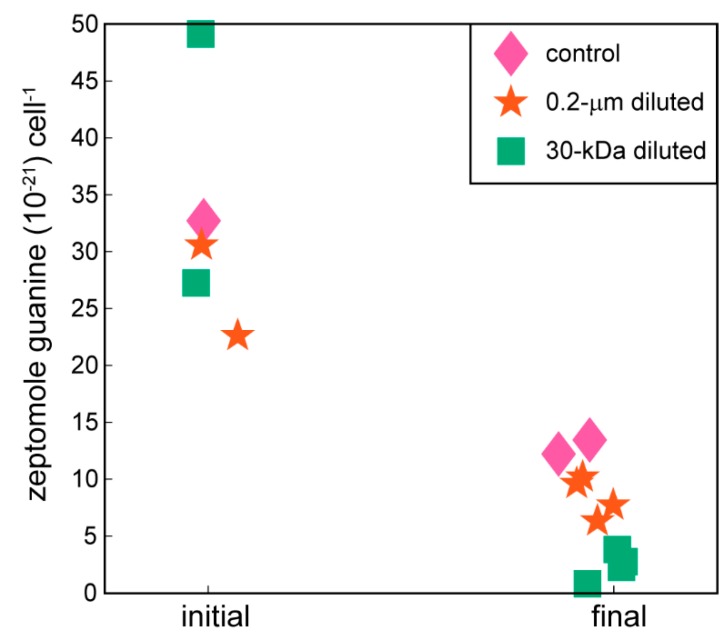
Cell-specific guanine concentrations at the initial and final time points of the experiment. Guanine is one example of a nucleic acid precursor measured with the targeted metabolomics method used in this project. Data are biological replicates from different bottles. The remaining nucleic acid precursors are plotted in Supplemental Figure S2.

Guanine, a nucleic acid precursor, showed decreases in cell-specific concentrations at the end of the 24-hour incubation period compared to the beginning of the experiment (Figure 2, one-way ANOVA, *p*-value = 0.0002). At the conclusion of the experiment, cell-specific guanine concentrations averaged a 5-fold decrease compared to the initial time points. The remaining nucleic acid precursors did not show statistically significant differences between the controls and the 0.2-µm diluted or 30-kDa diluted treatments (Supplemental Figure S2). 

**Figure 3:**
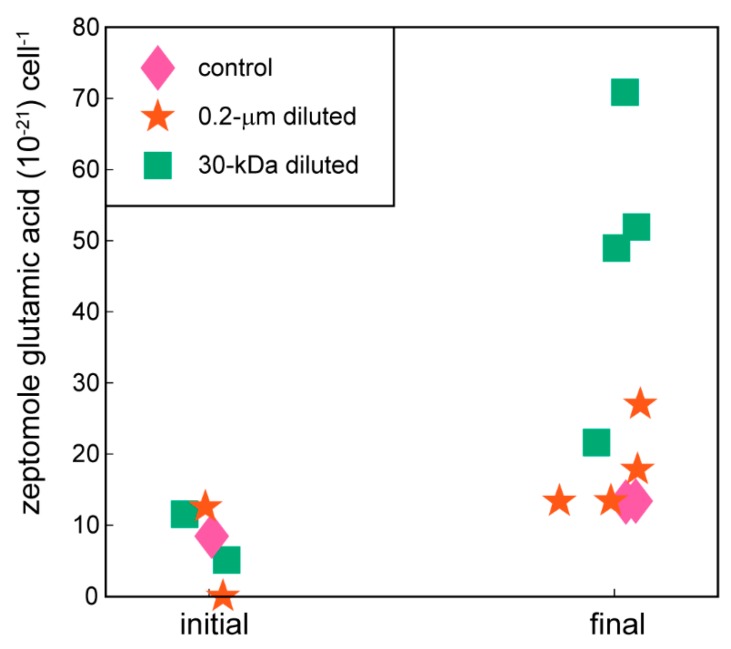
Cell-specific glutamic acid concentrations at the initial and final time points of experiment. Glutamic acid is one of the amino acids measured with the targeted metabolomics method used in this project. The remaining amino acids are plotted in Supplemental Figure S3.

**Figure 4:**
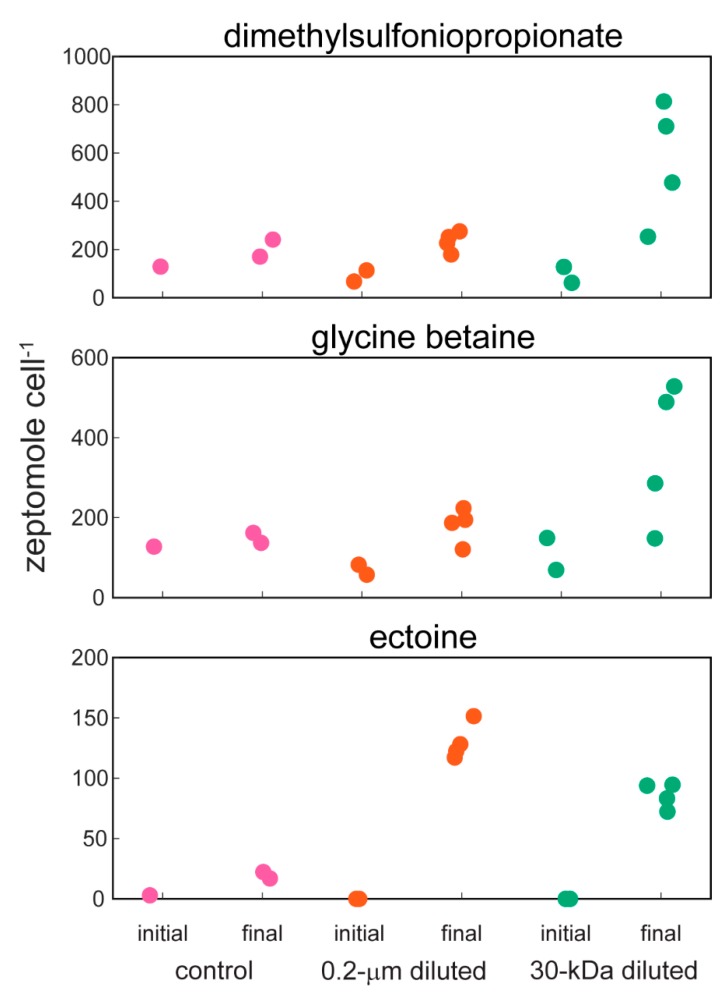
Cell-specific concentrations of three compounds that are known to act as compatible solutes within cells: dimethylsulfoniopropionate (DMSP), glycine betaine, and ectoine. Note the different *y*-axis scales for each compound. Data are jittered within each group for clarity.

Amino acids (Figure 3) and compatible solutes (Figure 4) accumulated within the intracellular metabolite pool and these changes were statistically significant for the amino acids phenylalanine and glutamic acid in the 30-kDa diluted treatment (one-way ANOVA, *p*-values < 0.05 followed post hoc by Dunnett’s test, Table 2); there was no significant difference between the 0.2-µm diluted treatment and the control. Other amino acids including arginine, glutamine, and proline showed similar, but not statistically significant, trends (Supplemental Figure S3). The accumulation of ectoine, a compatible solute, was also statistically significant in both the 0.2-µm diluted and 30-kDa diluted treatments (one-way ANOVA, *p*-value = 0.0001 followed post hoc by Dunnett’s test, Table 2, Figure 4). Glycine betaine and dimethylsulfoniopropionate (DMSP) are two additional compatible solutes that also showed similar, albeit not statistically significant, increases over the course of the incubation experiment (Figure 4).

### 2.3. Untargeted Metabolomics Data from LC FT-ICR MS Analysis 

The set of mzRT features (chemical features with unique combinations of *m/z* values and retention times) from the untargeted metabolomics methods provide a perspective on the complexity of organic matter found within microbial-sized cells living in seawater. The analysis of seawater using the untargeted metabolomics method requires an additional de-salting step, therefore some compounds that are measured with the targeted method may not show up in this analysis. The untargeted metabolomics analysis resulted in 2858 mzRT features in negative ion mode, and 65% of these mzRT features (1862 features) were observed in all fifteen of our samples. The average number of mzRT features at the beginning of the experiment was 2491 mzRT features, which is not statistically different from the average of 2400 mzRT features observed at the conclusion of the experiment (Wilcoxon rank sum test, *p*-value > 0.05). Within the mzRT features, compounds can be present as different adducts and with different numbers of ^13^C atoms. Using CAMERA [15], approximately 10% of the mzRT features were labeled as isotopologues or compounds present with different adducts. We did not remove the isotopologues or adducts from our analysis in order to allow us to analyze the complete set of mzRT features as we consider patterns in organic compounds found within the project. 

We used Weighted Correlation Network Analysis (WGCNA) to reduce the complexity of the untargeted metabolomics data. WGCNA is commonly used in the processing of genomics and transcriptomics data to find groups of genes that co-vary and thus may be regulated by similar factors. In metabolomics, WGCNA has been used to find groups of metabolites involved in the ripening of tomato plants [16] and to assess urinary metabolites that accumulate after traumatic injury [17]. 

**Figure 5:**
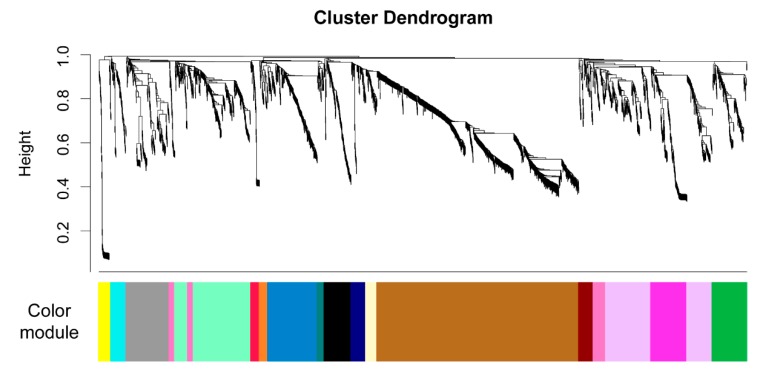
WGCNA analysis was used to group the mzRT features from the untargeted metabolomics data into modules. The figure shows the results from clustering 2858 mzRT features, and the color bar on the bottom indicates the arbitrary colors assigned to each module.

First, we calculated a signed network that connects mzRT features based on their shared patterns across the sample set. The result divides the untargeted metabolomics data into 17 modules as shown in Figure 5, and each module is summarized as an eigengene and assigned a color. The colors are arbitrary and are only used to simplify our discussion of each module within the WGNCA analysis. Each module is a set of connected mzRT features. We posit that each set of mzRT features are responding to the same environmental forcing, either because they represent a single type of organic compound or because they are handled by the *in situ* microbial community using a coherent set of biochemical pathways thereby resulting in similar concentration patterns throughout the experiment. The number of mzRT features in each color module ranged from 31 to 890, with three modules (brown, lavender, and mint) representing over 50% of the mzRT features (Figure 5). The remaining 1349 mzRT features were divided into 14 modules.

**Figure 6:**
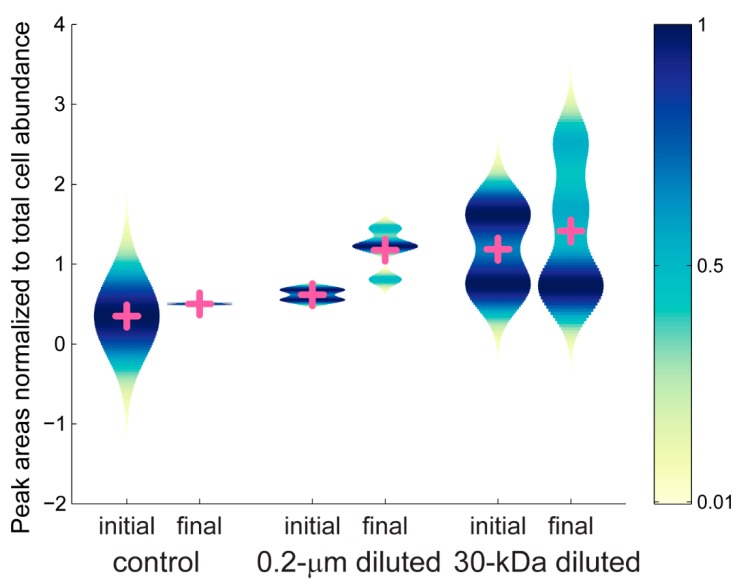
Average peak areas, normalized to total cell abundance, for the blue color module. Peak areas are scaled from 0 to 1. Mean values for each group are shown with the pink cross. Peak areas for all of the color groups are given in Supplemental Figure S4.

We further investigated the color modules to consider changes in the mzRT features during the experiment. We used violin plots to plot the average peak areas for each treatment within a given color group (Supplemental Figure S4). Violin plots allow us to visualize the probability density of the data at different values, in addition to presenting the mean values for each treatment. There are thousands of mzRT features in each sample and we use the violin plots to visually summarize the spread of peak areas within each treatment. Without the violin plots, we would have to present thousands of individual figures to show how each of the mzRT features present in a color module changed over time. The peak areas for the mzRT features show different patterns for each color module. For example, the mzRT features within the blue color module (Figure 6) reveal a slight increase in the mean peak areas by the final time point of the experiment in the control and the 30-kDa diluted treatments, and a larger increase in mean peak areas in the 0.2-µm diluted treatments. However, the range of average peak areas for the 30-kDa diluted treatment was broader at both the initial and the final time point compared to the range of peak areas in the 0.2-µm diluted samples. Furthermore, the 30-kDa diluted treatment at the initial time point presented a bimodal pattern in average peak area that was reduced to a skewed unimodal distribution by the final time point. A comparison across all the color modules (Supplemental Figure S4) revealed two general patterns. First, the maroon and red color modules showed the largest ranges of average peak areas, while the mint, grey, black, and pink color modules showed smaller differences in peak areas during the experiment. The peak areas can be used to infer the relative concentration of a metabolite, and increased variability in peak areas indicates increased shifts in metabolite concentrations. 

The next stage of the analysis relates the modules to external information. The WGCNA analysis calculates Pearson correlations between each eigengene and an external dataset (Supplemental Figure S5), here the set of organic compounds measured with the targeted metabolomics methods (Supplemental Table S1). Nine of the color modules were correlated with one or more organic compound (Table 3). The blue module was positively correlated with ectoine and proline and negatively correlated with ciliatine and guanine. With 218 mzRT features, this module was the largest module correlated with any targeted metabolomics data. Other known organic compounds correlated with a module include nucleic acid precursors (guanine, inosine, IMP; Supplemental Figure S2) and amino acids (glutamic acid, glutamine, phenylalanine; Supplemental Figure S3). However, the majority of the unknown mzRT features were not correlated to any known organic compounds, most notably the 890 mzRT features found in the largest module, the brown module (Table 3). Furthermore, nine of the organic compounds measured in the targeted metabolomics method, and found in at least three samples, were not correlated with any module.

## 3. Discussion

Marine microorganisms play a critical role in biogeochemical cycling because they consume, alter, and release organic matter. Metabolomics can play a central role in deciphering the chemical signals generated by microorganisms as they respond to variability in environmental conditions. However, characterizing the actions of a marine microbial community requires manipulating the microbial community and measuring resulting changes in the composition of organic matter. We used a combination of targeted and untargeted metabolomics approaches to consider short-term microbial responses to differences in mortality. In our study, only a small number of metabolites showed statistically significant changes in concentration although these changes were not accompanied by an increase in the number of microbial-sized cells. Lags in increasing microbial cell abundances have previously been observed as delays in the incorporation of radiolabeled tracers used to assess cell growth [6,18] and short-term changes in microbial physiology using fluorescent stains [19]. Our data suggest that delays in microbial growth may be caused by microorganisms expending energy altering their internal composition of organic matter prior to increasing cellular biomass. This de-coupling between changes in internal organic matter and microbial growth is important because it means we cannot assume metabolite levels remain static during different stages of microbial growth. 

Our original goal in this project was to assess how mortality may change the composition of organic matter in microbial cells. To address this question, we filtered water through membranes of different sizes to separate marine microorganisms from sources of mortality, viruses and/or protozoan grazers. In this experiment, the treatments with reduced levels of viral lysis and protozoan grazing (30-kDa diluted samples) revealed a greater change in the intracellular accumulation of metabolites compared to the response in the treatments with reduced levels of protozoan grazing only (0.2-µm diluted samples). The microbial community can release organic compounds while dilution experiments are established [20,21]. We posit that the additional filtration step required to generate the 30-kDa filtered seawater released organic compounds that enabled the microbial community to enhance their accumulation or consumption of select compounds such as amino acids or nucleic acid precursors. The concentration of these compounds would be on the zeptomolar scale we measured in the targeted metabolomics data and is, therefore, too small to measure with the high-temperature combustion method used to obtain the concentration of total organic carbon in these samples. Unlike Pasulka et al. [22], we did not observe differences in growth rates in the 30-kDa diluted water compared to the 0.2-µm diluted water. While increasing the duration of the experiment may have ultimately revealed differences in growth rates, there would also have been shifts in the diversity of the microbial community [11,23,24,25] which we opted to avoid by keeping the experiment to less than one day.

In all treatments, the amino acids increased and the nucleic acid precursors decreased by the conclusion of the incubation experiment. The intracellular accumulation of amino acids is not surprising given earlier observations that marine microorganisms can remove dissolved amino acids from seawater and thereby support increases in biomass [26]. At the same time, nucleic acid precursors decrease as cells are replicating nucleic acids thereby leaving fewer chemical precursors within the cells. The assimilation of amino acids, such as leucine, or nucleic acid precursors, e.g., thymidine, has long been used to quantify bacterial production in marine ecosystems (for a review, see [27]). The balance between the incorporation of leucine and thymidine has been connected to changes in the growth state of marine microorganisms [28,29] and higher leucine incorporation rates relative to thymidine incorporation rates are observed in metabolically active high nucleic acid cells [30]. Collectively, our current results and previous research emphasize that cells can accumulate amino acids relative to nucleic acid precursors even prior to changes in cellular abundance.

The intracellular accumulation of compatible solutes was an unexpected observation during this project. Compatible solutes, also called osmolytes or chemical chaperones, are used by microorganisms to balance intracellular and extracellular osmotic pressures. Yet, filtering seawater through a 0.2-um or 30-kDa filter would not have changed the osmotic pressure because the ions in seawater are smaller than these filters. Within cells, compatible solutes stabilize nucleic acids [31], and increase the stability [32] and fluidity [33] of bacterial membranes. Thus, an alternative hypothesis is that the accumulation of compatible solutes is a maintenance activity of the microbial community. Microbial cells partition energetic expenditures between growth and maintenance, and the balance of these processes determines how efficiently a microbial cell converts organic carbon into cellular biomass [34,35,36]. As a cell’s maintenance energy costs increase, there is less energy available to make new cells and a cell’s growth efficiency decreases. The lack of an increase in cell numbers during these 24-hour incubations is further evidence that the microbial cells are primarily expending energy for maintenance activities. 

A diverse collection of known compatible solutes are present within microorganisms [37] and we observed a surprising number of mzRT features that were correlated to known compatible solutes within our dataset. The most notable group was the blue color module with mzRT features that were positively correlated with ectoine and proline. One possibility is that these mzRT features are acting as compatible solutes within our cells, which would greatly expand the diversity of compounds acting as compatible solutes in marine systems. However, as we posit above, the accumulation of compatible solutes could be a maintenance activity in marine microorganisms. Therefore, these unknown mzRT features could represent organic compounds involved in a diverse array of cellular maintenance activities. Furthermore, we interpret the increased variability in peak areas in the 30-kDa treatment, relative to the 0.2-µm diluted treatment, as evidence of an increased range of maintenance activities in the 30-kDa treatment. This observation is consistent with previous observations of lower growth rates of microorganisms growing in 30-kDa filtered seawater [22]. Additional research will be needed to identify these unknown compounds and define their roles in cellular metabolism. Regardless, our observation of the key role of compatible solutes from both the targeted and untargeted metabolomics datasets aligns with genetic information indicating that an abundant marine microorganism, SAR11, dedicates a large component of its resources to the use of compatible solutes [38]. The combination of genomic data, our previous observations of increasing concentrations of compatible solutes in sinking marine particles [39], and the results from the current project collectively indicate that compatible solutes are critical component of organic matter within marine microorganisms.

Over three-quarters of the unknown mzRT features measured in this project were not correlated to a known organic compound. Many of these mzRT features showed little change in peak areas in response to the experimental manipulation or the time elapsed during the experiment. Therefore, these may be organic compounds that are consistently present within microbial cells, regardless of the environmental changes experienced by the cell. That these compounds were not correlated to any of our known compounds emphasizes that we still have much to learn about the metabolites found within marine microorganisms. 

Metabolomics can provide valuable insights into the actions of small microorganisms within marine ecosystems. Advances in instrumentation and the methods used to extract organic compounds from marine microorganisms now allow us to quantitatively assess organic compounds originating from a seawater matrix. However, future research projects should use additional biological replicates to better assess differences across treatments even though this requires larger volumes of water to be processed for each experiment. Computational advances, such as the WGCNA tool applied here, offer new ways to focus the large amounts of data generated by metabolomics experiments. Many of the challenges facing marine metabolomics have been observed in other fields; the large amount of time and effort required to identify novel compounds is the most obvious challenge. However, marine research faces additional challenges because we cannot establish controls that are free of organic carbon, nor can we separate marine microorganisms from the organic compounds they rely on for growth and energy. One unexpected outcome from the current project adds an interesting new observation to marine metabolomics: microorganisms can change their internal pool of organic matter without changing their abundance. As we move forward in marine metabolomics, we are excited by the challenge of continuing to investigate how marine microorganisms respond to changes in environmental conditions.

## 4. Materials and Methods 

### 4.1. Experimental Setup

Seawater for the incubation experiment was collected using 10 L Niskin bottles attached to a CTD/rosette system. The system includes a SBE9+ CTD equipped with dual SBE3T/SBE4C sensor systems for temperature and conductivity, a SBE43 oxygen sensor, and a Wet Labs combination fluorometer and turbidity sensor. Seawater from 70 m below the sea surface was collected off the northeastern corner of South America at 9.75° North, 55.3° West. Silicone tubing was used to collect water from the Niskins and the tubing was placed in the bottom of polycarbonate carboys in order to minimize turbulence during sample collection. The seawater was first filtered through a 0.2 μm Sterivex filter (Millipore) to obtain cell-free seawater. To obtain cell- and virus-free seawater, tangential flow filtration using a recirculating Prep/Scale tangential flow ultrafilter (Millipore) with a 30-kDa molecular mass cutoff was used.

Five different experimental treatments were established: (1) unfiltered, whole seawater as a control, (2) 20% whole seawater diluted with 0.2-µm filtered seawater, (3) 45% whole seawater diluted with 0.2-µm filtered seawater, (4) 20% whole seawater diluted with 30-kDa filtered seawater, (5) 45% whole seawater diluted with 30-kDa filtered seawater. Table 1 provides an overview of the experimental design for the project. There were three, 2 L polycarbonate bottles established for each treatment. One of the bottles was sampled immediately after the experiment was set up. The two remaining bottles were incubated for 24 h in an on-deck, flow-through incubator that allowed 10% of photosynthetically active radiation (PAR) to pass through its screening. As detailed below, the seawater in the bottles at the initial and final time points was sampled and processed to provide cell counts, total organic carbon concentrations, and compositional information on the intracellular organic compounds within microbial biomass.

### 4.2. Abundances of Microbial-Sized Cells 

Two methods were used to quantify the microbial cells within this experiment. Flow cytometry was used to obtain abundances of heterotrophic microorganisms and small autotrophic cells (phytoplankton). Seawater samples were fixed with 0.2% *w/v* paraformaldehyde (final concentration), placed in the dark for at least 10 min at room temperature to harden cells, and stored at −80 °C until sample processing. A Becton-Dickinson FACSCalibur flow cytometer was used to count small phytoplankton as described by Sherr et al. [40]; the presence of orange- or red-fluorescent pigments were used to distinguish *Synechococcus* and *Prochlorococcus*, respectively. Heterotrophic cells were counted after staining with a 1x working stock of SYBR Green I (Invitrogen, Carlsbad, CA) for 15 min [41]. To obtain the abundance of picoeukaryotes, cells were first preserved with 0.05% (final concentration) alkaline Lugol’s solution, followed by 0.1% (final concentration) sodium thiosulfate, and finally 2% (final concentration) of borate-buffered formalin. Samples were incubated at 4 °C for 24 h, stained with DAPI (25 μg mL^−1^ final concentration) for 10 min, and then filtered onto black 0.8 μm polycarbonate filters [42]. The filters were counted with epifluorescence microscopy. The total abundance of microbial-sized cells within each bottle was calculated as the sum of autotrophic and heterotrophic cells from the flow cytometry data and larger cells from the epifluorescence microscopy data. 

### 4.3. Concentration of Total Organic Carbon (TOC) 

TOC concentrations were measured on unfiltered seawater samples with a Shimadzu TOC-V_CSH_ total organic carbon analyzer using potassium hydrogen phthalate as a standard solution. TOC concentration was determined by subtracting the instrument blank area from the average peak area and dividing by the slope of the standard curve. The coefficient of variability between replicate injections was <1%. Comparisons to low carbon water and deep-sea reference water provided by Prof. D. Hansell (University of Miami) were made daily.

### 4.4. Processing of Seawater Samples for Intracellular Metabolomics

The remaining fluid in each bottle was filtered by gentle vacuum filtration onto 0.2-µm Omnipore (Millipore) filters. The filters were stored at −80 °C until extraction could occur in one batch for the entire experiment. The intracellular organic compounds, or metabolites, were extracted using a method modified from a protocol described by Rabinowitz and Kimball [43] and modified for seawater samples [8]. Briefly, the filter was extracted three times with ice-cold extraction solvent (acetonitrile:methanol:water with 0.1 M formic acid, 40:40:20). The combined extracts were neutralized with ammonium hydroxide and dried in a vacufuge. 

### 4.5. Targeted and Untargeted Mass Spectrometry

The organic matter extracts were analyzed using targeted and untargeted mass spectrometry methods described by Kido Soule et al. [8]. Briefly, the extracts for targeted mass spectrometry analysis were re-dissolved in 95:5 (*v/v*) water:acetonitrile with deuterated biotin (final concentration 0.05 µg mL^−1^) as an injection standard. These samples were then analyzed with a Synergi 4u Fusion – RP 80A 150 × 2.00 mm column (Phenomenex, Torrance, CA) coupled to a Thermo Scientific TSQ Vantage Triple Stage Quadrupole Mass Spectrometer. The chromatographic separation used a binary gradient with solvent A being water with 0.1% formic acid and solvent B being acetonitrile with 0.1% formic acid. Samples were run at 250 μL min^−1^ with 5% B for 0 to 2 min, ramp to 65% B from 2 to 20 min, ramp to 100% B from 20 to 25 min, and hold until 32.5 min. The column was re-equilibrated for 7 min between samples with 95% A. The samples were analyzed in random order with a pooled sampled run every six samples. The mass spectrometer was operated in selected reaction monitoring (SRM) mode; SRM parameters (s-lens, collision energy) for each target compound were optimized individually using an authentic standard. Two SRM transitions per compound were monitored for quantification and confirmation. Eight-point external calibration curves based on peak area were generated for each compound. The resulting data were converted to mzML files using the msConvert tool [44] and processed with MAVEN [45]. The detection limits for the targeted mass spectrometry data are provided in Johnson et al. [46]. Cell-specific concentrations were calculated as the zeptomoles (10^−21^ moles) of each organic compound divided by the total number of microbial cells. 

For untargeted analysis, the dried extracts were re-dissolved in 0.01 M hydrochloric acid and extracted using a 50 mg/1 cc PPL cartridge following the protocol of Dittmar et al. [47]. The additional extraction with the PPL cartridge will result in the loss of more polar compounds [46]. The resulting extracts were dried using a vacufuge and re-dissolved in 95:5 water:acetonitrile and deuterated biotin (final concentration 0.05 µg mL^−1^) and analyzed in negative ion mode with liquid chromatography (LC) coupled by electrospray ionization to a 7-Tesla Fourier-transform ion cyclotron resonance mass spectrometer (FT-ICR MS). LC separation was performed using the same conditions described above for the targeted analysis. Samples were analyzed in random order with a pooled sampled run every six samples in order to assess instrument variability. The resulting data were processed using the centWave algorithm [48] within XCMS [49] as described by Longnecker et al. [50]. The result is a list of mass-to-charge (*m/z*) ratios, retention times, and peaks areas in each sample. Here, we use the term ‘mzRT features’ to refer to chemical features with unique combinations of *m/z* values and retention times.

### 4.6. Statistics and Data Availability

We tested for statistically significant differences in cell-specific concentrations of organic compounds across the treatments (control, 0.2-µm diluted, 30-kDa diluted) at the final time point using a one-way ANOVA as implemented in MATLAB (Mathworks, Natick, MA). In each case, 0.2-µm diluted or 30-kDa diluted, the comparison was made to the whole seawater controls. Post-hoc multiple comparison tests were performed using Dunnett’s test to compare each treatment to the control. Prior to this analysis, we compared the bottles with 20% whole seawater (*n* = 3, including both initial and final bottles) to the bottles with 45% whole seawater (*n* = 3, including both initial and final bottles) and determined that there were no statistically significant differences (t-test, *p*-value > 0.05). For the remainder of the project, we grouped the data from the bottles with 20% whole seawater and 45% whole seawater. 

We used the R package Weighted Correlation Network Analysis (WGCNA [51]) to find groups of mzRT features that co-varied within the untargeted metabolomics data. WGCNA analysis can be briefly summarized in three steps. First, the analysis clusters mzRT features based on shared patterns in peak areas. The peak areas were log2 transformed prior to calculating the dissimilarity index using the Bray-Curtis metric and clustered using the Ward algorithm. The end result can be plotted as a dendrogram with all mzRT features.

Second, the mzRT features are divided into modules and a composite value is calculated for each module. The clusters were divided into modules using the blockwiseModules function with a Pearson correlation. We consider a signed network in order to allow both positive and negative correlations. We used the pickSoftThreshold function to determine that soft power = 12 with a scale-free threshold of 0.35 was optimal for our dataset. After dividing the mzRT features into modules, each module was labeled with an arbitrary color name. For each color module, the module eigengene was calculated. This eigengene is the first principal component of the eigenvectors used to define a color module, and can be considered an aggregate of the characteristics for each module.

Finally, WGCNA uses the average values of the eigengenes for each module to calculate correlations with an external dataset. In this project, the external dataset was the targeted metabolomics data. Correlations between the eigengenes and the known organic compounds were calculated using a Pearson correlation. Only correlations with *p*-values less than 0.05 were considered statistically significant. The results of this calculation allow us to connect the known organic compounds (metabolites from the targeted mass spectrometry analysis) with the unknown mzRT features measured in this project.

Violin plots were made in MATLAB using the distributionPlot function from Mathworks File Exchange. To make the plots, the average peak areas for each mzRT feature were first normalized by the cell abundance and then smoothed histograms were generated using a kernel smoothing function with a normal kernel. The cross represents the mean value for each subset of the data.

Targeted and untargeted metabolomics data for this project are available from MetaboLights (http://www.ebi.ac.uk/metabolights/) under accession number MTBLS461. The environmental data associated with the seawater used to establish this incubation experiment are available at BCO-DMO (http://www.bco-dmo.org/project/2204). The R Markdown notebooks used for the peak picking and WGCNA analysis are available at GitHub (http://github.com/KujawinskiLaboratory/DilutionExperiment). 

## Figures and Tables

**Table 1:** Experimental design and concentrations of total organic carbon (TOC) in each treatment at the initial and final time points. The control is 100% whole seawater, while the 0.2-µm and 30-kDa treatments were diluted with water passed through 0.2-µm and 30-kDa filters, respectively. The table provides the amount each treatment was diluted. * The initial TOC sample for the 0.2-µm treatment with 20% whole seawater was lost.

Treatment	% Whole Seawater	Time Point (# of Samples)	TOC (µM)
control	100%	initial (*n* = 1) final (*n* = 2)	76.9 80.3, 81.0
0.2-µm diluted	45%	initial (*n* = 1) final (*n* = 2)	82.0 77.5, 81.8
0.2-µm diluted	20%	initial (*n* = 1) final (*n* = 2)	* 84.7, 80.0
30-kDa diluted	45%	initial (*n* = 1) final (*n* = 2)	75.6 69.0, 80.8
30-kDa diluted	20%	initial (*n* = 1) final (*n* = 2)	81.7 77.7, 86.0

**Table 2:** Organic compounds that showed statistically significant differences from the control at the final time point of the incubation. The *p*-values are from the one-way ANOVA; the rows marked with an ‘x’ indicate metabolites with differences between each treatment and the control using the Dunnett’s post hoc multiple comparisons test. The sign (+ or -) indicates increase or decrease, respectively.

Metabolite	*p*-Value	0.2-µm Diluted (+/-)	30-kDa Diluted (+/-)
guanine	0.0002	x (-)	x (-)
glutamic acid	0.0263		x (+)
phenylalanine	0.0105		x (+)
ectoine	0.0001	x (+)	x (+)

**Table 3:** Summary of the 17 modules defined in the untargeted metabolomics data by the WGCNA analysis. The table provides the arbitrary color names and the number of mzRT features in each module. The positive and negative correlations were calculated between the eigengene defining each color module and the targeted metabolomics data.

Color (Arbitrary)	# mzRT Features	Positive Correlation	Negative Correlation
blue	218	ectoine proline	ciliatine guanine
magenta	159	glutamic acid	
green	153	xanthosine	glutamine
navy	65		D-ribose 5-phosphate syringic acid
maroon	64		inosine 5’-monophosphate
yellow	54	3-mercaptopropionic acid	
beige	49		NAD ^1^ pantothenic acid
red	38	proline	inosine
orange	37	glutamic acid phenylalanine	ciliatine guanine
brown	890		
lavender	310		
mint	309		
grey	189		
black	118		
pink	107		
cyan	67		
teal	31		

## Supplementary Materials

The following are available online at https://www.mdpi.com/2218-1989/10/3/105/s1, Figure S1: Nucleic acid precursors, Figure S2: Changes in amino acid concentrations, Figure S3: Average peak areas for each color module, and Figure S4: Heatmap with correlations between the targeted and untargeted metabolomics data.
