# Supplementary material for: Intracellular Metabolites in Marine Microorganisms during an Experiment Evaluating Microbial Mortality
Source: Metabolites. 2020 Mar 12;10(3):105. doi: 10.3390/metabo10030105 (PMC7142611; doi:10.3390/metabo10030105)
Supplement: Supplementary file 1 [file metabolites-10-00105-s001.pdf]

Appendix A

Supplemental Table S1.

Table S1. Compounds from the targeted mass spectrometry analysis that were found in three or more samples. A total of 70 unique organic compounds were in the analytical method. Based on the results from the WGCNA analysis, most of the compounds were correlated to unknown mzRT features and this is noted in the table.

Compound	Correlated to Unknown mzRT Features?
3-hydroxybenzoic acid	
3-mercaptopropionic acid	yes
4-hydroxybenzoic acid	
5'-methylthioadenosine	
arginine	
ciliatine	yes
citric acid	
desthiobiotin	
dimethylsulfoniopropionate	
D-Ribose 5-phosphate	yes
ectoine	yes
glutamic acid	yes
glutamine	yes
glycine betaine	
guanine	yes
inosine	yes
inosine 5'-monophosphate	yes
NAD[1]	yes
pantothenic acid	yes
phenylalanine	yes
proline	yes
salicylic acid	
syringic acid	yes
xanthosine	yes

[1]Nicotinamide adenine dinucleotide

Supplemental Figure S1

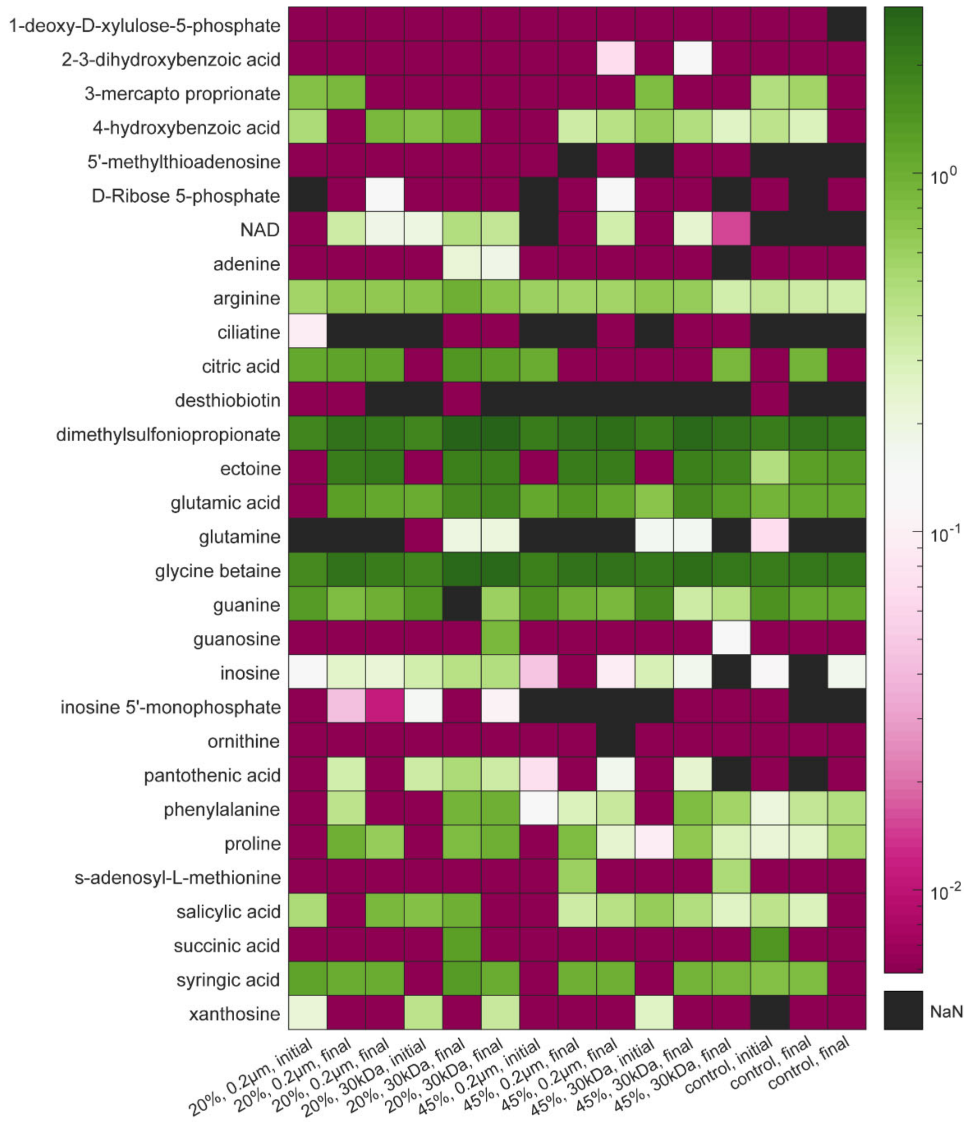

Figure S1. Cell-specific concentrations of metabolites measured with the targeted metabolomics method and present in at least three samples. Data are log10-transformed and plotted as a heat map.

Supplemental Figure S2

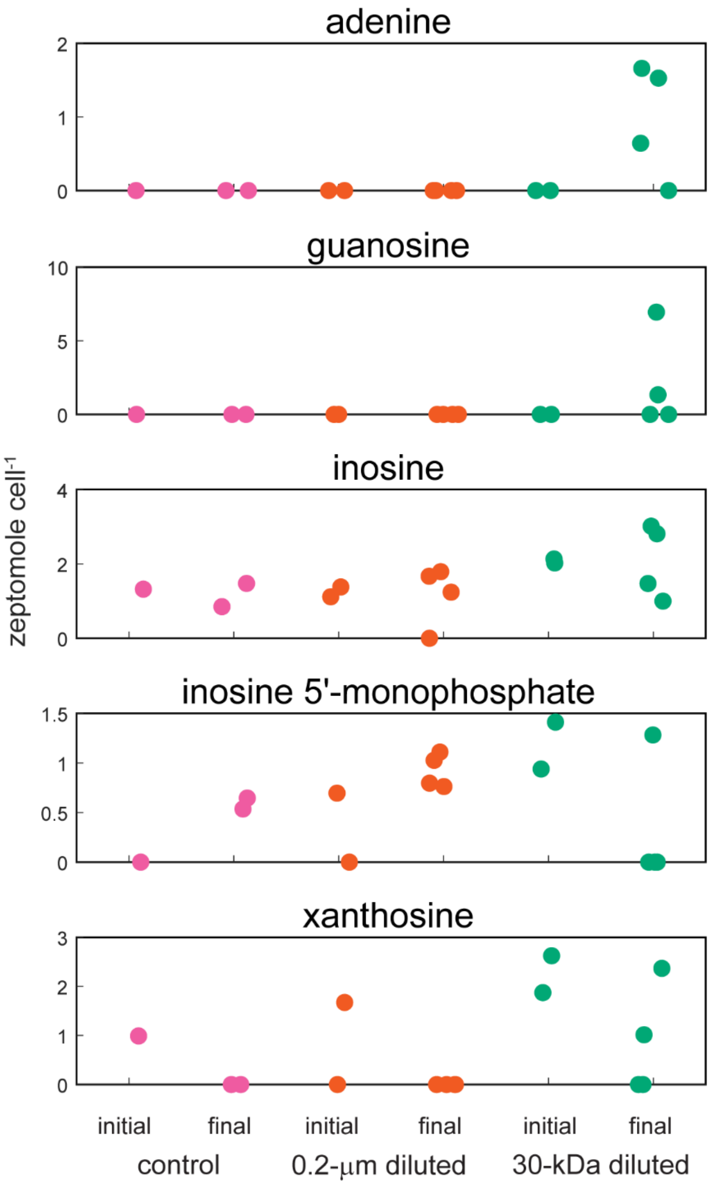

Figure S2. Nucleic acid precursors observed in the control, the 0.2-μm diluted treatment, and the 30-kDa diluted treatment. Cell-specific concentrations are shown as zeptomole $cell^{-1}$ for the initial and final time points for each treatment. Data for guanine, another nucleic acid precursor, are given in Figure 2. For the control n = 1 at the initial time point, and n = 2 at the final time point. For the 0.2-μm and 30-kDa diluted treatments, n = 2 at each initial time point, and n = 4 at each final time point.

Supplemental Figure S3

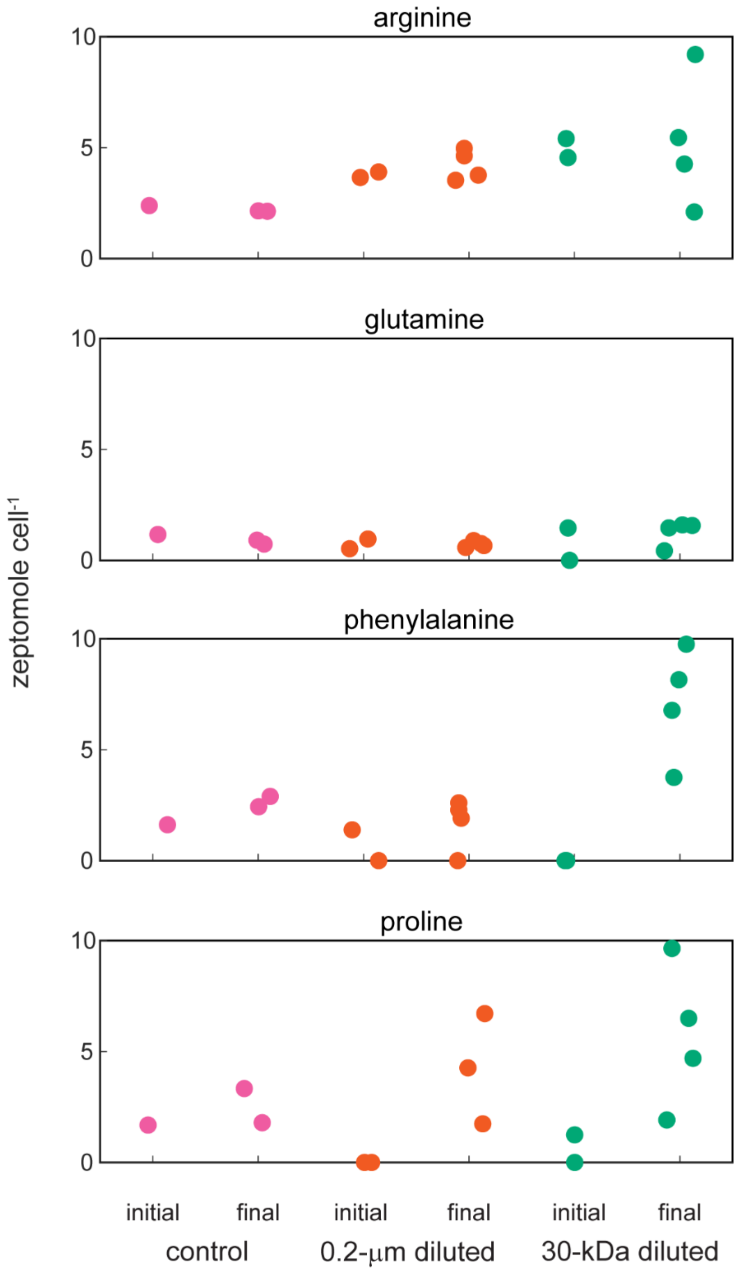

Figure S3. Changes in amino acids observed in the control, the 0.2-μm diluted treatment, and the 30-kDa diluted treatment. Cell-specific concentrations are shown as zeptomole cell^{-1} for the initial and final time points for each treatment. Data for glutamic acid, another amino acid, are given in Figure 3. For the control n = 1 at the initial time point, and n = 2 at the final time point. For the 0.2-μm and 30-kDa diluted treatments, n = 2 at each initial time point, and n = 4 at each final time point.

Supplemental Figure S4

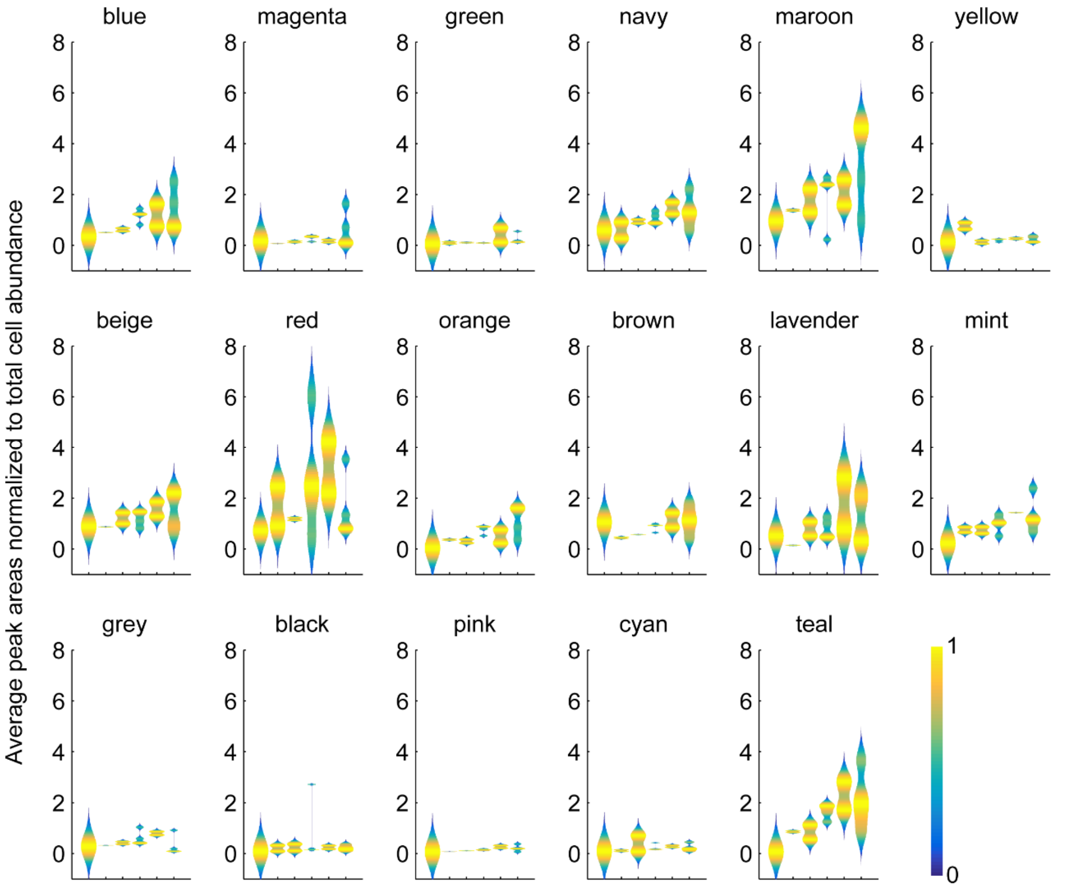

Figure S4. Average peak areas, normalized to cell abundance, for each color module. The name of the color module is given at the top. There are six groups in each color module that are plotted in the following order: control, initial; control, final; 0.2 µm diluted, initial; 0.2 µm diluted, final; 30 kDa diluted, initial; 30 kDa diluted, final. The peak areas are scaled from 0 (lowest, blue) to 1 (highest, yellow), and the color bar in the lower right of the figure shows the range. The blue color group is also plotted as Figure 6.

Supplemental Figure S5

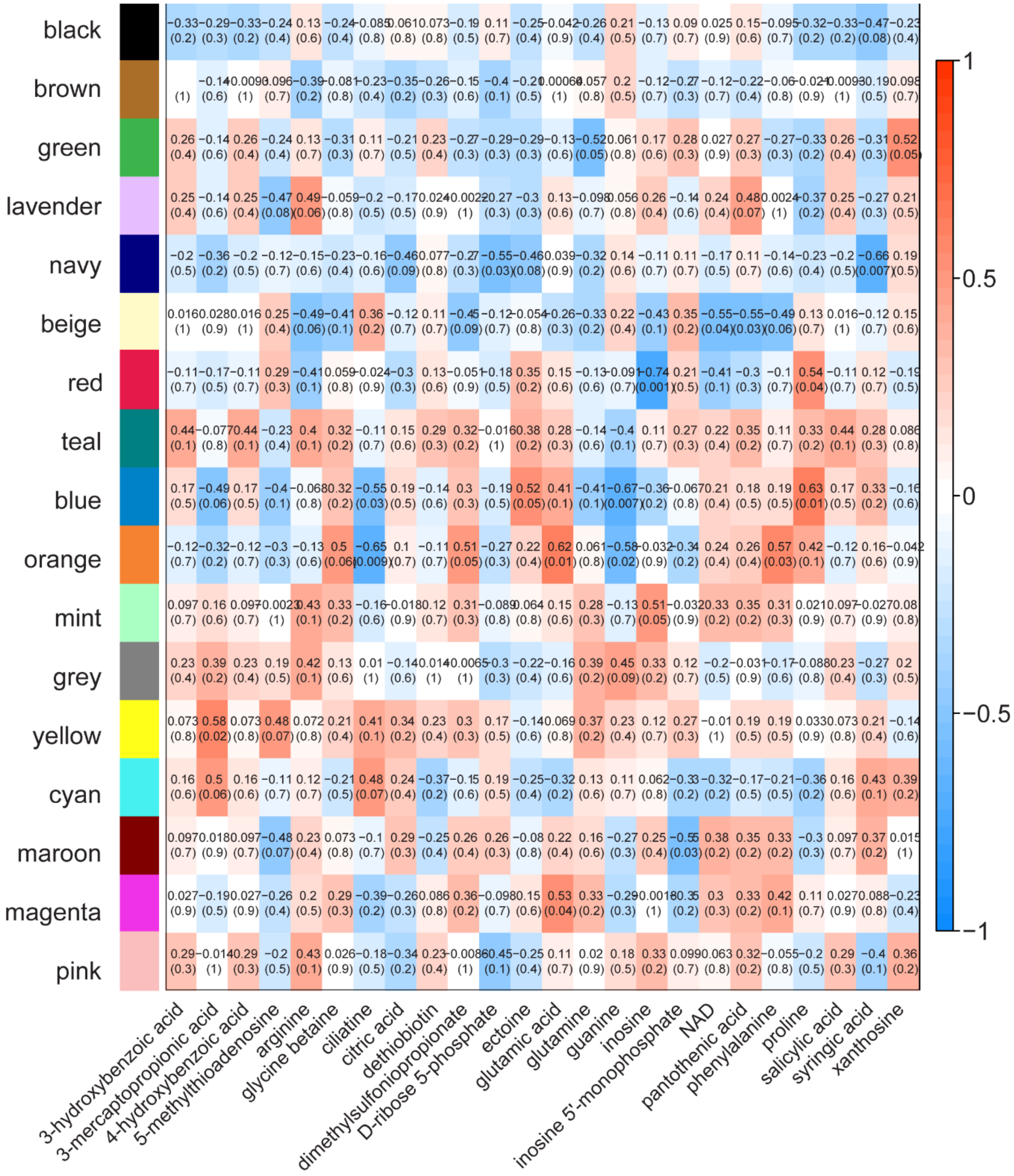

Figure S5. Heatmap showing the positive and negative correlations between the unknown mzRT features from the untargeted mass spectrometry analysis and the known metabolites from the targeted metabolomics analysis. The unknown mzRT features are presented in groups as color modules based on the output of the WGCNA analysis described in the text. The colors are arbitrarily assigned to each group, the number of mzRT features in each group are given in Table 3. Each red-to-blue block contains the value for the Pearson correlation (and the corresponding *p*-value); the colorbar on the right provides the color scale for the Pearson correlation coefficients. NAD is nicotinamide adenine dinucleotide.
